# Dynamic *vs* Static ABCG2 Inhibitors to Sensitize Drug Resistant Cancer Cells

**DOI:** 10.1371/journal.pone.0015276

**Published:** 2010-12-07

**Authors:** Hui Peng, Jing Qi, Zizheng Dong, Jian-Ting Zhang

**Affiliations:** 1 Department of Pharmacology and Toxicology, Indiana University School of Medicine, Indianapolis, Indiana, United States of America; 2 IU Simon Cancer Center, Indiana University School of Medicine, Indianapolis, Indiana, United States of America; University of Washington, United States of America

## Abstract

Human ABCG2, a member of the ATP-binding cassette transporter superfamily, plays a key role in multidrug resistance and protecting cancer stem cells. ABCG2-knockout had no apparent adverse effect on the development, biochemistry, and life of mice. Thus, ABCG2 is an interesting and promising target for development of chemo-sensitizing agents for better treatment of drug resistant cancers and for eliminating cancer stem cells. Previously, we reported a novel two mode-acting ABCG2 inhibitor, PZ-39, that induces ABCG2 degradation in addition to inhibiting its activity. In this manuscript, we report our recent progresses in identifying two different groups of ABCG2 inhibitors with one inhibiting only ABCG2 function (static) and the other induces ABCG2 degradation in lysosome in addition to inhibiting its function (dynamic). Thus, the inhibitor-induced ABCG2 degradation may be more common than we previously anticipated and further investigation of the dynamic inhibitors that induce ABCG2 degradation may provide a more effective way of sensitizing ABCG2-mediated MDR in cancer chemotherapy.

## Introduction

ABCG2 is a member of the ATP-binding cassette (ABC) transporter superfamily and over-expression of ABCG2 has been shown to cause multidrug resistance (MDR) in model cancer cell lines and to correlate with poor prognosis in both adult and childhood leukemia and breast cancer patients (for reviews see [1], [2], [3]). Unlike most other members of the ABC transporter superfamily such as P-glycoprotein (MDR1/ABCB1), ABCG2 is considered as a half transporter consisting of one nucleotide-binding domain (NBD) at amino terminus and one membrane-spanning domain (MSD) at carboxyl terminus. It has, thus, been thought to exist and function as a homo-dimer. However, recent evidence showed that ABCG2 may exist and function as a higher order of oligomer consisting of 8–12 identical subunits [4], [5] and the oligomerization sites are likely located in the MSD [6].

In the process of aiming to sensitize MDR mediated by ABCG2, a number of ABCG2 inhibitors have been recently discovered [7], [8], [9], [10], [11], [12] in addition to the previously identified ones such as Fumitremorgin C (FTC) (for a review see [2]). One of these ABCG2 inhibitors, PZ-39, was very effective and distinctive from others such as FTC with the ability to cause lysosome-dependent degradation of ABCG2 protein [7].

To further determine if inhibitor-induced ABCG2 degradation is unique to PZ-39, we tested other ABCG2 inhibitors generated during our initial screening which led to identification of PZ-39. We found two types of ABCG2 inhibitors with one inhibiting ABCG2 activity only (static) and the other inhibiting ABCG2 activity as well as inducing ABCG2 degradation via lysosome (dynamic). These findings suggest that inhibitor-induced ABCG2 degradation in lysosome may be more common than it has previously been anticipated and further investigating the dynamic inhibitors that induce ABCG2 degradation in lysosome may provide a more effective way of sensitizing ABCG2-mediated MDR in cancer chemotherapy.

## Results

### Two types of ABCG2 inhibitors

**Figure 1:**
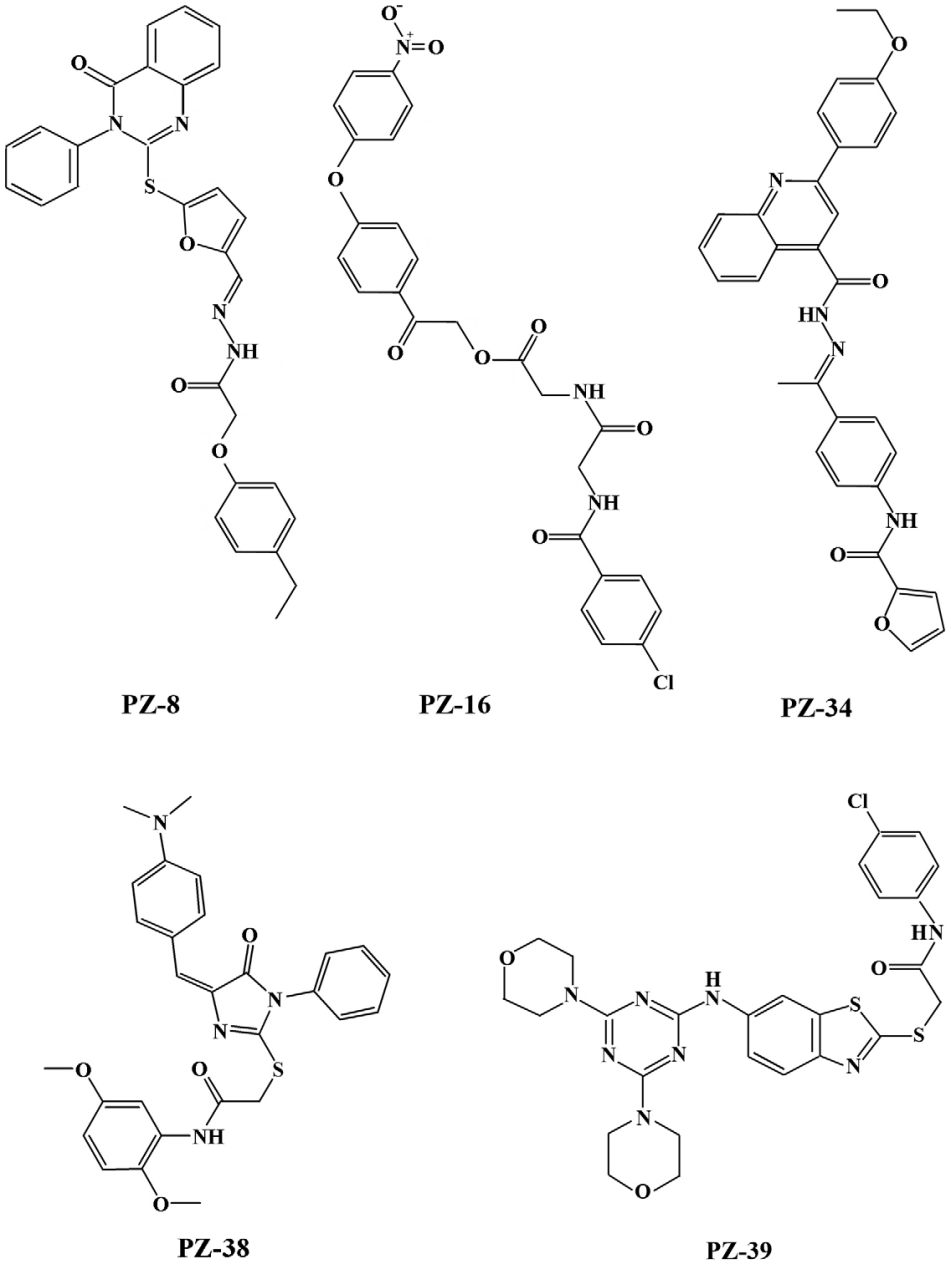
Structures of PZ-8, 16, 34 and 38 in comparison with PZ-39. The chemical structures are shown for PZ-8, (12E)-N'-((5-(3,4-dihydro-4-oxo-3-phenylquinazolin-2-ylthio)furan-2-yl)methylene)-2-(4-ethylphenoxy)acetohydrazide; PZ-16, 2-(4-(4-nitrophenoxy)phenyl)-2-oxoethyl2-(2-(4- chloro benzamido)acetamido)acetate; PZ-34, (E)-2-(4-ethoxyphenyl)-N'-(1-(4-(furan-2-carboxamido) phenyl)ethylidene)quinoline-4-carbohydrazide; PZ-38, (N-(2,5-dimethoxyphenyl)-2-({4-[4-(dimethylamino)benzylidene]-5-oxo-1-phenyl-4,5-dihydro-1H-imidazol-2-yl}sulfanyl)acetamide); and PZ-39 (N-(4-chlorophenyl)-2-[(6-{[4,6-di(4- morpholinyl)-1,3,5- triazin-2-yl] amino}-1,3-benzothiazol-2-yl)sulfanyl]acetamide).

**Figure 2:**
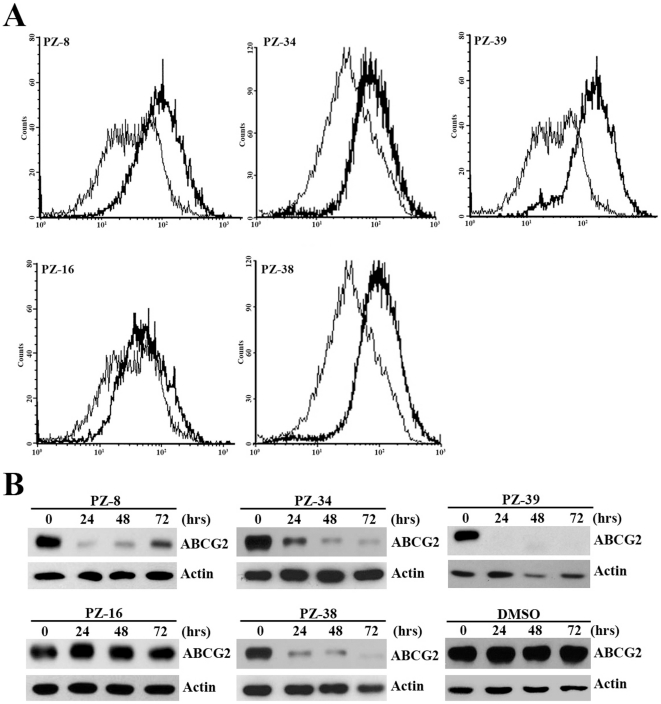
Effect of PZ compounds on mitoxantrone accumulation and ABCG2 expression. A, mitoxantrone accumulation. HEK293/ABCG2 cells were incubated with mitoxantrone for 30 min in the presence of DMSO (thin line) or 10 µM PZ compounds (thick line) followed by FACS analysis of mitoxantrone level. B, ABCG2 expression. HEK293/ABCG2 cells were incubated with 3.3 µM PZ compounds or DMSO control for various times followed by collection of cells and Western blot analysis of ABCG2 probed with monoclonal antibody BXP-21.

Previously, we reported that the rational screening of representatives of different types of compound library from Specs (www.specs.net) led to identification of a two-mode acting ABCG2 inhibitor PZ-39 [7]. During the initial screening, several other ABCG2 inhibitors, which are structurally different from PZ-39 and its derivatives (Fig. 1), were also identified and their activity to inhibit ABCG2-mediated drug efflux has been confirmed using HEK293 cells with over-expression of ectopic ABCG2 (HEK293/ABCG2) (Fig. 2A). To determine if these inhibitors also posses the two-mode acting property, we first tested the effect of these inhibitors on ABCG2 expression using Western blot analysis. As shown in Fig. 2B, three of the four new inhibitors (PZ-8, 34, and 38) along with PZ-39 inhibit ABCG2 expression while PZ-16 does not. Together with our previous finding that FTC inhibits only ABCG2 activity [7], we conclude that there are likely two types of ABCG2 inhibitors with one inhibiting only ABCG2 activity while the other inhibiting both the activity and expression of ABCG2.

### Suppression of ABCG2 expression by the known and existing ABCG2 inhibitors

**Figure 3:**
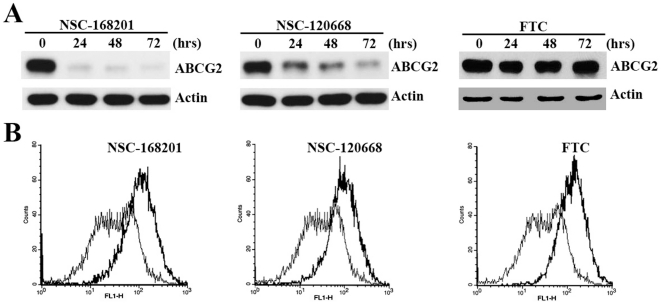
Effect of NSC compounds on ABCG2 expression and mitoxantrone efflux. A, ABCG2 expression. HEK293/ABCG2 cells were treated with 10 µM each of the known ABCG2 inhibitors NSC-168201, NSC-120668, or FTC for various times followed by Western blot analysis of ABCG2 expression probed with monoclonal antibody BXP-21. B, mitoxantrone accumulation. HEK293/ABCG2 cells were incubated with mitoxantrone for 30 min in the presence of DMSO (thin line) or 10 µM NSC-168201, NSC-120668, or FTC (thick line) followed by FACS analysis of intracellular mitoxantrone level.

The above results suggest that the inhibitor-induced suppression of ABCG2 expression may be more common than anticipated. To further test this possibility, we investigated the effect of two other published ABCG2 inhibitors (NSC-168201 and NSC-120668) [12] on ABCG2 expression using Western blot analysis. As shown in Fig. 3A, both NSC-168201 and NSC-120668 effectively suppress ABCG2 expression. However, the control ABCG2 inhibitor FTC does not although all three inhibitors effectively enhance mitoxantrone accumulation in HEK293/ABCG2 cell lines (Fig. 3B). Thus, we conclude that the inhibitor-induced suppression of ABCG2 expression may be more common than it has been anticipated and there are possibly two groups of ABCG2 inhibitors.

### Specific effect of PZ-34 and PZ-38 on ABCG2-mediated mitoxantrone efflux

**Figure 4:**
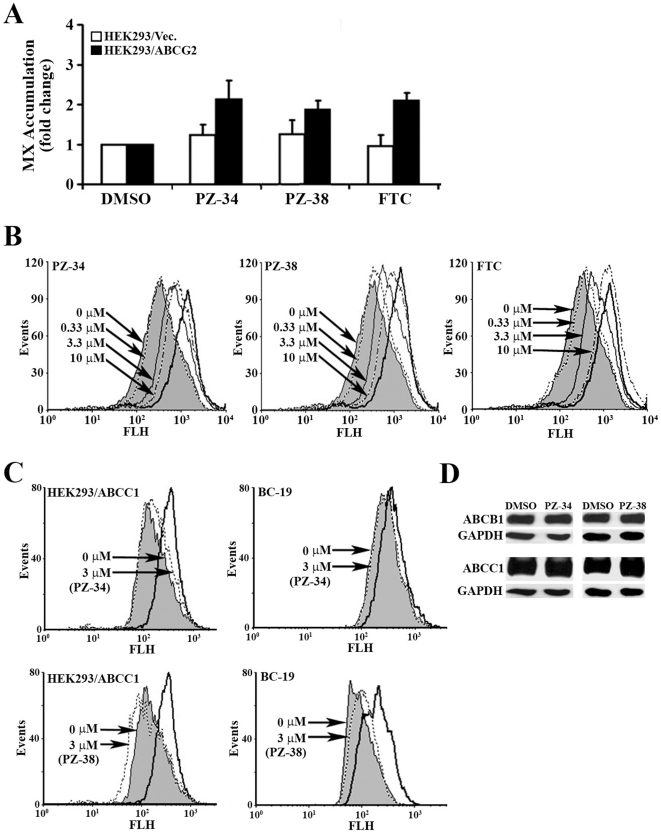
PZ-34 and PZ-38 inhibition of ABCG2-mediated mitoxantrone efflux. A, mitoxantrone accumulation. HEK293/Vec or HEK293/ABCG2 cells were incubated with mitoxantrone for 30 minute in the presence of DMSO control, or 3 µM of PZ-34, PZ-38 or FTC control. The data are means ± SD from three independent experiments. B, dose response of PZ-34, PZ-38, and FTC control in restoring mitoxantrone accumulation in HEK293/ABCG2 cells. The thick line shows the level of mitoxantrone accumulation in HEK293/Vec cells, serving as a maximum accumulation control. C, effect on ABCB1 and ABCC1-mediated Adriamycin efflux. HEK293 cells with ABCC1 over-expression (HEK293/ABCC1) and BC19 cells with ABCB1 over-expression were incubated with Adriamycin in the absence (gray area) or presence (dotted line) of 3.8 µM PZ-34 or PZ-38 followed by FACS analysis. The thick line indicates the maximum level of accumulation in cells transfected with vector control. D, effect on ABCB1 and ABCC1 expression. HEK293/ABCC1 and BC19 cells with ABCB1 over-expression were treated with 10 µM PZ-34 or PZ-38 for 3 days followed by Western blot analysis of ABCB1 using monoclonal antibody C219 and ABCC1 using monoclonal antibody MRPr1. GAPDH was used as a loading control.

To further investigate if these new inhibitors suppress ABCG2 expression by inducing ABCG2 degradation in lysosome, we chose to focus on PZ-34 and PZ-38 and first performed a detailed analysis of their effects on drug accumulation. As shown in Fig. 4A, both PZ-34 and PZ-38 at ∼4 µM increase mitoxantrone accumulation to a similar degree as the well-established ABCG2 inhibitor FTC in HEK293/ABCG2 cells. These compounds, however, have no significant effect on mitoxantrone accumulation in the control cells-transfected with vector (HEK293/Vec), indicating that the effect of PZ-34 and PZ-38 on mitoxantrone accumulation is likely via inhibiting ABCG2. We then tested the dose response of PZ-34 and PZ-38 in inhibiting ABCG2-mediated mitoxantrone efflux in HEK293/ABCG2 cells using flow cytometry. As shown in Fig. 4B, the intracellular mitoxantrone level is much less in HEK293/ABCG2 cells compared with HEK293/Vec cells due to ABCG2-mediated efflux. Addition of PZ-34 and PZ-38 increases the intracellular accumulation of mitoxantrone in a dose-dependent manner similar as FTC.

To determine the specificity of PZ-34 and PZ-38, we tested their effect on drug efflux mediated by two other ABC transporters that are known to cause MDR, ABCB1 and ABCC1, using MCF7 cells-transfected with ABCB1 (BC19) [13] and HEK293 cells-transfected with ABCC1 (HEK293/ABCC1) [14], [15]. However, we found no effect of these compounds on the activity of ABCB1 and ABCC1 in decreasing Adriamycin accumulation (Fig. 4C). Both PZ-34 and PZ-38 also do not affect the expression of ABCB1 and ABCC1 (Fig. 4D). Thus, PZ-34 and PZ-38 may be specific to ABCG2 and do not affect drug efflux mediated by two other major ABC transporters.

### Effect of PZ-34 and PZ-38 on ABCG2-mediated multidrug resistance

**Figure 5:**
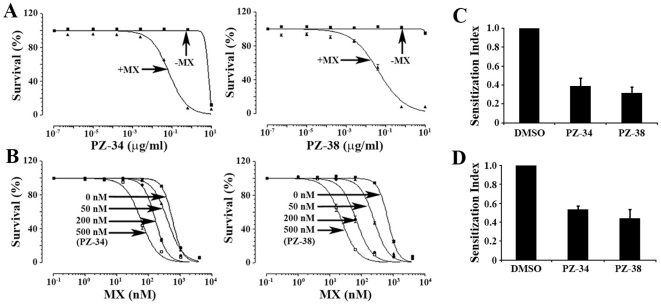
Effect of PZ-34 and PZ-38 on sensitizing drug resistance. A, potency of PZ-34 and PZ-38 in reversing mitoxantrone resistance. HEK293/ABCG2 cells were treated without or with 0.1 µM (IC_10_) mitoxantrone in the absence or presence of different concentrations of PZ-34 or PZ-38 followed by SRB assay. B, sensitization index of PZ-34 and PZ-38 in HEK293/ABCG2 cells. HEK293/ABCG2 cells were treated with various concentrations of mitoxantrone in the absence or presence of different concentrations of PZ-34 or PZ-38 followed by SRB assay. C and D, sensitization index of PZ-34 and PZ-38 in drug-selected MCF7/AdVp3000 cells. MCF7/AdVp3000 cells were treated with various concentrations of mitoxantrone (C), or Adriamycin (D) in the presence of DMSO (vehicle) or 500 nM of PZ-34 and PZ-38 followed by MTT assay. Sensitization index was calculated using IC_50_ of the anticancer drugs in the absence or presence of PZ-34, PZ-38, or FTC. The data shown are mean ± SD of three independent experiments.

To determine if both PZ-34 and PZ-38 have the ability to sensitize ABCG2-mediated drug resistance, we performed analyses of the effects of these compounds on survival of HEK293/ABCG2 cells in the absence or presence of mitoxantrone. As shown in Fig. 5A, both PZ-34 and PZ-38 alone have no effect on the survival of HEK293/ABCG2 cells at concentrations less than 10 µg/ml. Their IC_50_ were estimated to be ∼12.9 and >20 µM, respectively (Table 1). Thus, both PZ-34 and PZ-38 have little cytotoxicity to HEK293/ABCG2 cells at concentrations less than 10 µM, suggesting that they may not act on other essential molecules with high affinity for cell survival.

**Table 1:** Potency index of PZ-34 and PZ-38 for sensitization of mitoxantrone resistance in HEK293/ABCG2 cells.

	IC_50_ (µM) of inhibitor		
	Inhibitor alone	Inhibitor + MX^a^	Potency Index^b^
PZ-34	12.94±0.15	0.071±0.01	182
PZ-38	>20	0.045±0.031	>444
FTC	24.8±1.4	0.326±0.086	76

a. MX = mitoxantrone at 100 nM that produces ≤10% inhibition of growth (IC_10_).

b. Potency Index = ratio of inhibitor IC_50_ in the absence and presence of mitoxantrone at low concentration (<IC_10_).

We next determined the effect of PZ-34 and PZ-38 on sensitizing cellular response to 0.1 µM mitoxantrone which alone produces less than 10% inhibition of growth of HEK293/ABCG2 cells. As shown in Fig. 5A, both PZ-34 and PZ-38 at a low concentration range sensitize these cells to mitoxantrone. At about 1.2 µM, both PZ-34 and PZ-38 assist 0.1 µM mitoxantrone to completely inhibit cell growth. The IC_50_ of PZ-34 and PZ-38 in sensitizing mitoxantrone resistance were calculated to be 71 and 45 nM, respectively (Table 1). We also tested PZ-8 and PZ-16 and found that the IC_50_ of these compounds alone is ∼20 µM and their IC_50_ in sensitizing mitoxantrone resistance of MCF7/AdVp3000 cells are ∼80 and ∼70 nM, respectively. The IC_50_ of FTC in sensitizing mitoxantrone resistance, on the other hand, is much higher at 326 nM (Table 1).

Next, we examined the dose response of PZ-34 and PZ-38 in sensitizing ABCG2-mediated mitoxantrone resistance using HEK293/ABCG2 cells. As shown in Fig. 5B, the IC_50_ of mitoxantrone decreases dramatically in the presence of PZ-34 and PZ-38. At 50 nM, PZ-34 and PZ-38 significantly reduce the IC_50_ of mitoxantrone with a sensitization index of 1.6 and 2.2, respectively (Table 2). At 500 nM, the sensitization index of PZ-34 and PZ-38 are 8.7 and 14.3, respectively (Table 2). On the other hand, the sensitization index of FTC at 500 nM is only 3.9 (Table 2). These results show that PZ-34 and PZ-38 are very potent novel ABCG2 inhibitors.

**Table 2:** Sensitization index of PZ-34 and PZ-38 on mitoxantrone resistance in HEK293/ABCG2 cells.

	IC_50_ (nM) of Mitoxantrone and SI^a^					
	50 nM	SI	200 nM	SI	500 nM	SI
PZ-34	346.6±58.8	1.6	149.7±13.4	3.7	63.7±10.7	8.7
PZ-38	250.0±38.2	2.2	91.6±24.2	6.0	38.7±13.3	14.3
FTC	467.8±42.6	1.2	254.7±7.9	2.2	143.1±39.3	3.9

a. SI = Sensitization Index, determined by dividing median IC_50_ of mitoxantrone (551 nM) in the presence of vehicle (0.1% DMSO) by IC_50_ of mitoxantrone in the presence of inhibitor.

To further investigate if PZ-34 and PZ-38 can reverse ABCG2-mediated MDR in a drug resistant cancer cell line, we used the drug-selected breast cancer cell line MCF7/AdVp3000 which over-expresses ABCG2 and tested an additional anticancer drug substrate of ABCG2, Adriamycin. As shown in Fig. 5C, both PZ-34 and PZ-38 at 500 nM drastically reduce the resistance of MCF7/AdVp3000 to both Adriamycin and mitoxantrone.

### PZ-34 and PZ-38 do not affect ABCG2 oligomerization

Previously, it has been shown that ABCG2 may exist and function as a homo-oligomer [4], [6]. To examine if PZ-34 and PZ-38 possibly inhibit ABCG2 oligomerization, co-immunoprecipitation of two differentially tagged ABCG2 was performed as previously described [4] following a 6-hr treatment with PZ-34 and PZ-38 at 3.3 µM. However, no effect of PZ-34 and PZ-38 on ABCG2 co-immunoprecipitation was found (see supplemental Fig. S1), suggesting that PZ-34 and PZ-38, similar to PZ-39 [7], may not affect ABCG2 oligomerization. However, due to the limitation of co-immunoprecipitation, these inhibitors may affect dimerization which can not be revealed by co-immunoprecipitation due to the existence of at least 3 different interaction sites of the oligomeric ABCG2 [4], [6].

### Effect of PZ-34 and PZ-38 on the half-life of ABCG2

As discussed above, both PZ-34 and PZ-38 suppressed ABCG2 expression (Fig. 2). To rule out the possibility that this suppression is due to inhibition of gene expression, we performed real time RT-PCR analysis. As shown in Fig. S2, the steady state levels of ABCG2 mRNA are the same between control and compound treatment groups and, thus, eliminating the possibility that these compounds affect the transcription or stability of ABCG2 mRNAs.

**Figure 6:**
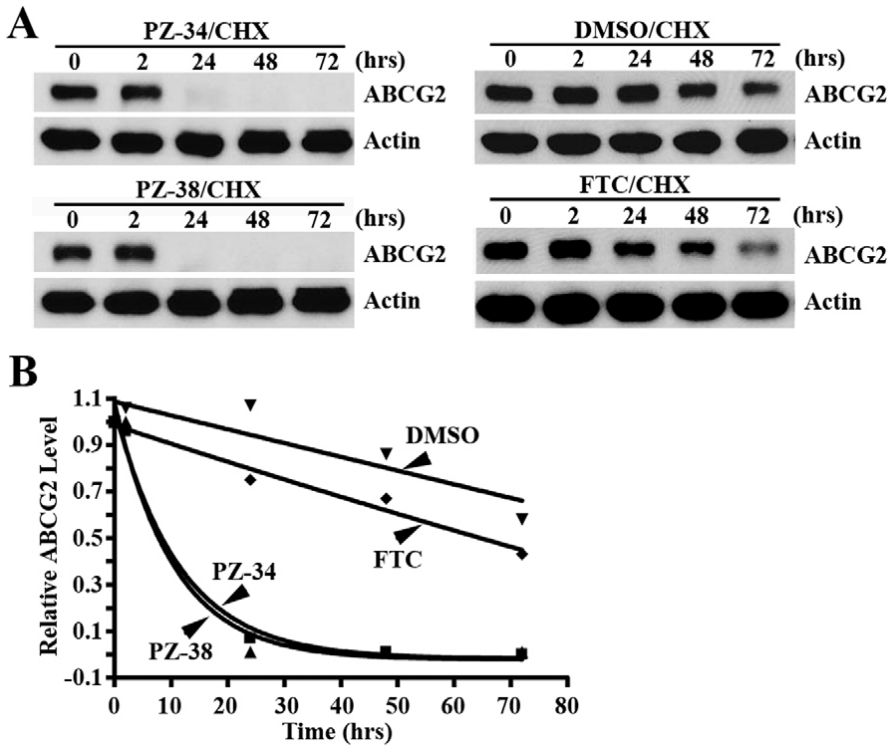
Effect of PZ-34 and PZ-38 on ABCG2 stability. A, Western blot analysis. HEK293/ABCG2 cells were first treated with 5 µg/ml cycloheximide (CHX) followed by addition of 3 µM of PZ-34, PZ-38, FTC, or DMSO control for various times. The cells were then harvested for Western blot analysis of ABCG2 probed with monoclonal antibody BXP-21. Actin was used as a loading control. B, half-life of ABCG2. ABCG2 levels on Western blot as shown in panel A were determined using Scion Image and plotted against time of treatment. Data shown are mean± S.D of three experiments.

To determine if the suppressed expression of ABCG2 is due to inhibitor-induced degradation as we previously observed with PZ-39 [7], we examined the effect of PZ-34 and PZ-38 on the half-life of ABCG2 in HEK293/ABCG2 cells by pre-treating cells with cycloheximide, which inhibits elongation during protein synthesis, followed by treatment with PZ-34 and PZ-38 for various times. As shown in Fig. 6A, the loss of ABCG2 in cells treated with a combination of cycloheximide and PZ-34 or PZ-38 is much faster than that of the control treatment with DMSO vehicle or FTC control. The half-life of ABCG2 in the presence of PZ-34 and PZ-38 is estimated to be ∼8 hrs whereas it is stable with an estimated half-life more than 60 hrs in the control treatment with FTC or DMSO (Fig. 6B). Thus, PZ-34 and PZ-38 likely accelerates the degradation of ABCG2 protein similarly as PZ-39 [7].

### PZ-34 and PZ-38 induce ABCG2 degradation in lysosome

**Figure 7:**
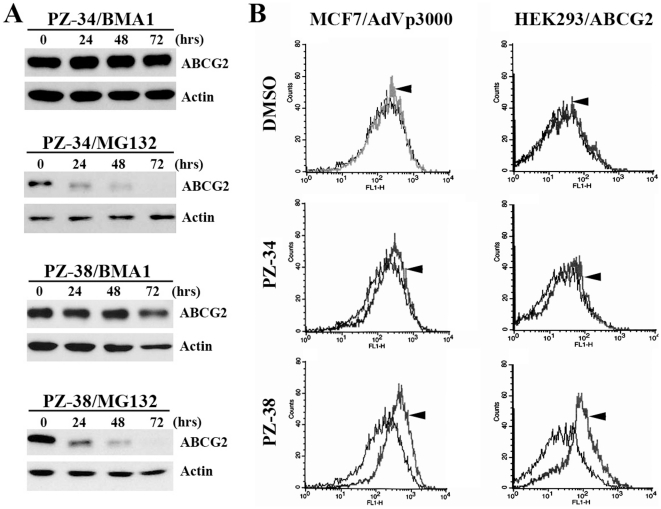
PZ-34 and PZ-38-binding induced ABCG2 degradation and conformational change. A, ABCG2 degradation in lysosomes. HEK293/ABCG2 cells were treated with 3 µM of PZ-34 or PZ-38 in the absence or presence of 10 nM Bafilomycin A_1_ (BMA1) or 2 µM MG-132 for various times. The cells were then harvested for Western blot analysis of ABCG2 expression probed with monoclonal antibody BXP-21. Actin was used as a loading control. B, ABCG2 conformational changes. HEK293/ABCG2 and MCF7/AdVp3000 cells were treated with 10 µM of PZ-34, PZ-38, or DMSO vehicle control followed by staining with monoclonal antibody 5D3 and FACS analysis. Arrowhead indicate the PZ-34 and PZ-38 treated groups.

It has been reported previously that wild-type and correctly-folded ABCG2 proteins are degraded in lysosome whereas the mutant and misfolded proteins are involved in ubiquitin-mediated degradation in proteasome [16]. In addition, we found previously that PZ-39 causes ABCG2 degradation via lysosome-mediated degradation [7]. To determine if PZ-34 and PZ-38 cause ABCG2 degradation via lysosome or proteasome, we used Bafilomycin A_1_, an inhibitor of protein degradation in lysosome, and MG-132, a proteasome inhibitor as previously described [7]. As shown in Fig. 7A, pre-treatment of cells with Bafilomycin A_1_ inhibits PZ-34 and PZ-38-induced ABCG2 degradation whereas pre-treatment with MG-132 does not. Thus, likely PZ-34 and PZ-38 also induce ABCG2 degradation in lysosome, same as PZ-39 [7].

### PZ-34 and PZ-38 bind to ABCG2

It has been reported previously that the monoclonal antibody 5D3 binds to ABCG2 on cell surface more readily upon binding of inhibitors to ABCG2 possibly due to inhibitor-induced conformational changes of ABCG2 [7], [12], [17]. To determine if PZ-34 and PZ-38 potentially bind to ABCG2, we performed staining analyses of ABCG2 using 5D3 in the presence or absence of PZ-34 and PZ-38. As shown in Fig. 7B, both PZ-34 and PZ-38 cause an increase in 5D3 staining in both MCF7/AdVp3000 and HEK293/ABCG2 cells, indicating that both PZ-34 and PZ-38 may bind to ABCG2 and the binding leads to the conformational change of ABCG2.

## Discussion

In the present study, we show that there are possibly two groups of ABCG2 inhibitors and the inhibitor-induced ABCG2 degradation in lysosome may be more common than previously anticipated. We also show that PZ-34 and PZ-38 are potent ABCG2 inhibitors. Although PZ-34 and PZ-38 are structurally different from the previously identified ABCG2 inhibitor, PZ-39, they appear to have similar mechanism of action by inhibiting ABCG2 function and by accelerating ABCG2 degradation in lysosome.

Among many ABCG2 inhibitors previously identified, few are known to be specific to ABCG2 and none has been investigated to show if they could accelerate ABCG2 degradation in lysosome. In this and our previous studies [7], we found that FTC did not affect ABCG2 expression whereas both NSC-168201 and NSC-120668 did. In the four new ABCG2 inhibitors (PZ-8, 16, 34, and 38) tested in this study, three suppressed ABCG2 expression while the other did not. Taken together, we believe that there are two groups of ABCG2 inhibitors with one inhibiting only ABCG2 activity and the other also suppressing ABCG2 degradation in addition to inhibiting ABCG2 function. We name these inhibitors as static and dynamic inhibitors, respectively.

It is currently unknown what fundamental differences between these two groups of inhibitors cause the difference in their mechanism of action. It is, however, tempting to speculate that they bind to two different sites on ABCG2. Binding to either site will cause conformational changes of ABCG2 which lead to inhibition of ABCG2 activity. However, binding to one of the sites will also facilitate ABCG2 endocytosis and degradation in lysosome. The change of ABCG2 conformation by PZ-34 and PZ-38 detected using the monoclonal antibody 5D3 suggests that PZ-34 and PZ-38 directly bind to ABCG2 although their binding sites are currently unknown. Since FTC also causes conformational change but does not accelerate ABCG2 degradation, PZ-34 and PZ-38 likely do not bind to the similar site as FTC. Previously, it has been shown that agonist binding accelerated endocytosis and degradation of β_2_-adrenergic receptor in lysosome [18], supporting the above hypothesis. Although unlikely, it is also possible that the dynamic ABCG2 inhibitors may have off-target effect that activates the upstream pathways involved in ABCG2 degradation. Regardless, these possibilities need to be tested in future in-depth studies.

Previously, it has been shown that ABCG2 degradation occurs mainly via two different mechanisms. While correctly folded wild type ABCG2 are mainly degraded via lysosome [16], the mutant proteins are degraded by proteasome via a quality control mechanism [16], [19], [20]. It appears that the quality control mechanism occurs at the ER right after the synthesis of ABCG2 and normal degradation of the wild type proteins may occur through endocytosis of ABCG2 from plasma membranes. Currently, it is not yet known if the dynamic inhibitor-induced degradation of ABCG2 occurs by trafficking to lysosome from plasma membranes via endocytosis and/or from ER membranes immediately following their synthesis.

Although it is currently unknown if PZ-34 and PZ-38 are specific to ABCG2, our results show that they do not affect ABCB1 and ABCC1 function and expression. Thus, PZ-34 and PZ-38 are more specific to ABCG2 than some of the previously identified ABCG2 inhibitors such as the known ABCG2 inhibitor GF120918 which appears to inhibit ABCB1 and/or ABCC1 equally well [2], [21]. We also found that both PZ-34 and PZ-38 are not cytotoxic with a concentration up to 10 µg/ml, suggesting that these ABCG2 inhibitors probably do not bind to and inhibit other cellular proteins with high affinity that are essential for cellular survival. However, more studies are needed to investigate the specificity of PZ-34 and PZ-38 and to determine if they bind to and inhibit other members of the human ABC transporter family.

The fact that PZ-34 and PZ-38 have no cytotoxicity to HEK293 cells at concentrations less than 10 µM and can effectively reverse MDR suggests that the window of therapeutic index of these compounds are large. An ideal chemo-sensitizer is that it should not be toxic itself. Clearly, PZ-34 and PZ-38 satisfy this requirement in the in-vitro studies. However, it is not known if these compounds are toxic and effective in reversing MDR in vivo, which need to be evaluated in future studies using animal models.

## Materials and Methods

### Materials

Monoclonal antibody BXP-21 against ABCG2, anti-Myc and anti-HA antibodies were from ID Labs, Cell Signaling, and Roche, respectively. Monoclonal antibody C219 and MRPr1 were purchased from Kamiya Biomedical Company. Biotin-conjugated 5D3 antibody and Phycoerythrin-Streptavidin conjugates were from eBiosciences. All electrophoresis reagents, protein concentration assay kit, and polyvinylidene difluoride membranes were purchased from Bio-Rad. FTC, Adriamycin, mitoxantrone, camptothecin, dithiothreitol, Sulforhodamine B (SRB), and Triton X-100 were from Sigma. Compounds PZ-8, PZ-16, PZ-34, PZ-38, and PZ-39 were purchased from SPECS. NSC-168201 and NSC-120668 were gifts from National Cancer Institute. Protein-G PLUS-Agarose and SYBR Green PCR Master Mix were from Santa Cruz Biotechnology and Applied Biosystems, respectively. LipofectAMINE Plus and G418 were from Invitrogen. Cell culture medium IMEM and DMEM were from BioSource International and Media Tech, respectively. All other chemicals were of molecular biology grades from Sigma or Fisher Scientific.

### Cell culture, treatments and lysate preparations

Human breast cancer cell line MCF7 and its derivative lines BC19 (a gift from Julie Horton at National Institute of Environmental Health Sciences) and MCF7/AdVp3000 (a gift from Susan Bates at National Cancer Institute), HEK293/ABCC1 [14], HEK293/vector [14], HEK293/ABCG2 [4] were cultured as previously described [4], [13], [14], [22]. For analysis of ABCG2 degradation, HEK293/ABCG2 cells were first treated with 10 nM Bafilomycin A_1_ or 2 µM MG132 for 24 hrs followed by treatment with 3 µM PZ-34 or PZ-38 for various times and collection of cell lysates. For the determination of ABCG2 half-life, HEK293/ABCG2 cells were treated with 5 µg/ml cycloheximide, 3 µM PZ-34 or PZ-38 for various times followed by collection of cells. Lysate preparation was performed as described previously [22].

### Western blot, immunoprecipitation, and FACS analyses

Western blot, immunoprecipitation, and FACS analyses of drug accumulation were performed exactly as we previously described [4], [6]. To determine the conformational change of ABCG2 following treatment with ABCG2 inhibitors, HEK293/ABCG2 cells were incubated with 10 µM PZ-34 or PZ-38 at 37°C for 30 min before incubating with biotin-conjugated 5D3 antibody (1∶100 dilution) for 2 hrs. The cells were then washed 3 times and incubated with Phycoerythrin-Streptavidin for 30 min followed by washing and analysis using FACS.

### Cytotoxicity assay

Cytotoxicity was determined using SRB and MTT assays as previously described [6], [22], [23]. The effect of ABCG2 inhibitors on drug resistance was determined by exposing cells to a range of concentrations of anticancer drugs in the absence or presence of different concentrations of ABCG2 inhibitors. The potency and sensitization index of ABCG2 inhibitors were calculated as follows: 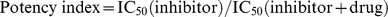


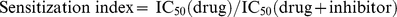



### Real time RT-PCR

RNA extraction and real-time RT-PCR were performed as we described previously [22]. The sequences of ABCG2 primers are 5′-GGCTTTCTACCTGCACGAAAACCAGTTGAG-3′ (forward) and 5′-ATGGCGTTGAGACCAG-3′ (reverse). The sequences of GAPDH primers are 5′-AAGGACTCATGACCACAGTCCAT-3′ (forward) and 5′-CCATCACGCCACAGTTTCC-3′ (reverse). The relative ABCG2 RNA level (2^ΔCT^) treated with inhibitors was expressed as percentage of the control (in the presence of 0.1% DMSO) where ΔCT (threshold cycle)  =  (CT_ABCG2_-CT_GAPDH_).

## Supporting Information

Figure S1 **Effect of PZ-34 and PZ-38 on ABCG2 dimerization/oligomerization.** HEK293 cells co-transfected with Myc- and HA-tagged ABCG2 were exposed to 3.3 µM PZ-34, PZ-38, or DMSO control for 6 hrs and cell lysates were subjected to immunoprecipitation with anti-Myc or anti-HA monoclonal antibody followed by western blot analysis probed using anti-HA and anti-Myc antibodies.
(JPG)

Figure S2 **Effect of PZ-34 and PZ-38 on ABCG2 mRNA level.** HEK293/ABCG2 cells were treated with DMSO vehicle, PZ-34, or PZ-38 for various times and harvested for RNA preparation and real-time RT-PCR analysis. Data shown are mean ± SD from three independent experiments.
(JPG)
