# Supplementary figures and images for: Dynamic vs Static ABCG2 Inhibitors to Sensitize Drug Resistant Cancer Cells
Source: PLoS One. 2010 Dec 7;5(12):e15276. doi: 10.1371/journal.pone.0015276 (PMC2998423; doi:10.1371/journal.pone.0015276)

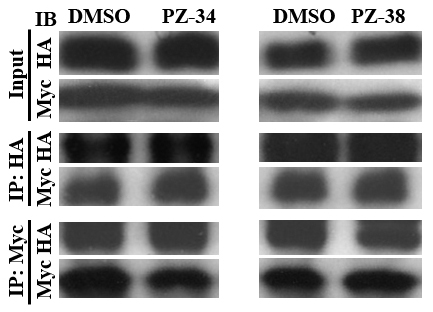

Supplement: Figure S1 — Effect of PZ-34 and PZ-38 on ABCG2 dimerization/oligomerization. HEK293 cells co-transfected with Myc- and HA-tagged ABCG2 were exposed to 3.3 µM PZ-34, PZ-38, or DMSO control for 6 hrs and cell lysates were subjected to immunoprecipitation with anti-Myc or anti-HA monoclonal antibody followed by western blot analysis probed using anti-HA and anti-Myc antibodies. (JPG) [file pone.0015276.s001.jpg]

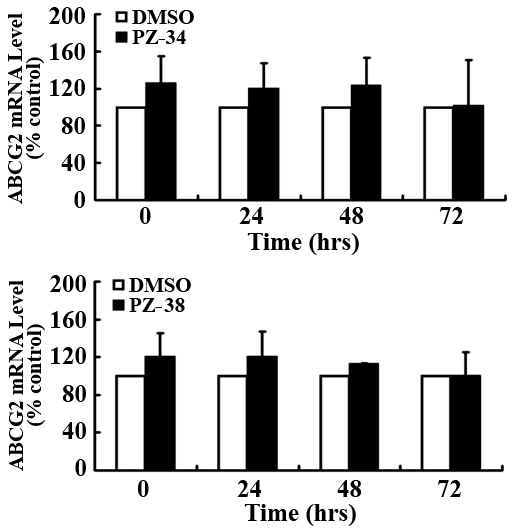

Supplement: Figure S2 — Effect of PZ-34 and PZ-38 on ABCG2 mRNA level. HEK293/ABCG2 cells were treated with DMSO vehicle, PZ-34, or PZ-38 for various times and harvested for RNA preparation and real-time RT-PCR analysis. Data shown are mean ± SD from three independent experiments. (JPG) [file pone.0015276.s002.jpg]
